# An Update on MyoD Evolution in Teleosts and a Proposed Consensus Nomenclature to Accommodate the Tetraploidization of Different Vertebrate Genomes

**DOI:** 10.1371/journal.pone.0001567

**Published:** 2008-02-06

**Authors:** Daniel J. Macqueen, Ian A. Johnston

**Affiliations:** Gatty Marine Laboratory, School of Biology, University of St Andrews, St Andrews, Fife, Scotland; Baylor College of Medicine, United States of America

## Abstract

**Background:**

MyoD is a muscle specific transcription factor that is essential for vertebrate myogenesis. In several teleost species, including representatives of the Salmonidae and Acanthopterygii, but not zebrafish, two or more MyoD paralogues are conserved that are thought to have arisen from distinct, possibly lineage-specific duplication events. Additionally, two MyoD paralogues have been characterised in the allotetraploid frog, *Xenopus laevis*. This has lead to a confusing nomenclature since MyoD paralogues have been named outside of an appropriate phylogenetic framework.

**Methods and Principal Findings:**

Here we initially show that directly depicting the evolutionary relationships of teleost MyoD orthologues and paralogues is hindered by the asymmetric evolutionary rate of Acanthopterygian MyoD2 relative to other MyoD proteins. Thus our aim was to confidently position the event from which teleost paralogues arose in different lineages by a comparative investigation of genes neighbouring *myod* across the vertebrates. To this end, we show that genes on the single *myod*-containing chromosome of mammals and birds are retained in both zebrafish and Acanthopterygian teleosts in a striking pattern of double conserved synteny. Further, phylogenetic reconstruction of these neighbouring genes using Bayesian and maximum likelihood methods supported a common origin for teleost paralogues following the split of the Actinopterygii and Sarcopterygii.

**Conclusion:**

Our results strongly suggest that *myod* was duplicated during the basal teleost whole genome duplication event, but was subsequently lost in the Ostariophysi (zebrafish) and Protacanthopterygii lineages. We propose a sensible consensus nomenclature for vertebrate *myod* genes that accommodates polyploidization events in teleost and tetrapod lineages and is justified from a phylogenetic perspective.

## Introduction

The myogenic regulatory factors (MRFs) are a family of vertebrate proteins (individually MyoD, Myf5, Mrf4 and Myog), that are potent transcription factors for muscle genes [1]. This potency lies in two conserved domains, the basic region and helix-loop-helix (i.e. the bHLH). Ubiquitously expressed E-proteins share the bHLH and dimerise with MRFs through their respective HLH's and the resulting complex then binds via the basic regions to a conserved motif called the e-box, which is widely conserved in the regulatory regions of muscle genes [2]. MyoD, Myf5 and Mrf4 have overlapping but developmentally distinct functions in the specification and differentiation of myoblasts, whereas Myog and Mrf4 activate and maintain the terminal differentiation of muscle [1], [3]. The four MRFs are ancient paralogues and thus arose through gene duplication events [4], [5]. It has been hypothesised that the entire genome of the lineage leading to modern vertebrates has duplicated twice during evolution [6]. This is thought to explain the prevalence of vertebrate gene families with up to four members relative to basal deuterostome and protostome animals [7], a pattern nicely recapitulated by the MRFs. It was proposed that a single ancestor gene, currently conserved from fruit fly to jellyfish to tunicates, was first duplicated to produce the ancestor genes to MyoD/Myf5 and Mrf4/Myog which subsequently duplicated again, resulting in the current MRFs [4], [5]. The teleost genome went through whole genome duplication (WGD) again around 320–350 Mya [8], [9], meaning most species can potentially have two paralogues of any Sarcopterygian gene. Furthermore, more recent polyploidization events within specific vertebrate lineages [reviewed by 10], [11] means further copies may have been generated, resulting in additional levels of complexity when resolving phylogenetic relationships of paralogues and orthologues.

In most diploid tetrapods, including birds, mammals, the frog *Xenopus tropicalis* as well as teleosts of the Ostariophysi superorder, MyoD is represented by a single gene (Table 1). The allotetraploid frog, *X. laevis* has two differentially expressed MyoD paralogues that were originally named Xlmf1 and Xlmf25 [12] (Table 1). Teleost species of the Acanthopterygii superorder also have two differentially expressed paralogues originally denoted MyoD1 (orthologous to the single *myod* gene of the Ostariophysi [5]) and MyoD2 [13] (Table 1). Additionally, two salmonid MyoD duplicates were characterised in rainbow trout (*Oncorhynchus mykiss*) and named MyoD and MyoD2 [14]. More recently a third salmonid MyoD sequence was characterised [5]. In Atlantic salmon (*Salmo salar*) the three paralogues have distinct embryonic expression fields that together recapitulate the expression of zebrafish MyoD [5]. A maximum likelihood phylogenetic reconstruction revealed that all salmonid paralogues were co-orthologues of Ostariophysi-MyoD/Acanthopterygii-MyoD1 and arose independently of Acanthopterygian MyoD2 [5]. However, Acanthopterygian MyoD2 proteins appeared as an outgroup to both teleost and tetrapod MyoD sequences with 100% branch confidence, which does not reflect either a common teleost origin or a lineage specific event. This tree topology is probably an artefact of the asymmetric evolution of MyoD2 relative to other MyoD proteins, obscuring its true phylogenetic position by long branch attraction (LBA) or mutational saturation within the alignment. Here, we initially attempted to correct the suspected aberrant topology, by using several methods of phylogenetic reconstruction, taking actions to reduce LBA and remove mutational saturation. Our next aim was to investigate the chromosomal regions proximal to vertebrate *myod* genes using a comparative-genomic and phylogenetic approach to confidently establish the extent of the duplication event from which Acanthopterygian MyoD1 and MyoD2 paralogues arose. Interestingly, this approach provided strong evidence that a *myod*-containing chromosome duplicated in a common teleost ancestor, probably during the WGD event of basal teleost evolution [9], which directly contradicted the majority of tree topologies retrieved by direct phylogenetic reconstruction. These results allow us to advocate the use of a sensible nomenclature consensus for vertebrate *myod* genes that accommodates polyploidzation events in teleosts, amphibians and other non-diploid vertebrates.

**Table 1:** Details of teleost MyoD sequences, including their current designation, Genbank accession number/Ensembl gene ID and suggested correct designations (proposed consensus nomenclature)

Taxa	Species	Current designation	Sequence	GenBank accession	Ensembl gene ID	Suggested designation
**Tetrapoda**	*Homo sapiens*	MyoD	complete	CAA40000	ENSG00000129152	MyoD
	*Mus musculus*	MyoD	complete	X56677	ENSMUSG00000009471	MyoD
	*Gallus gallus*	MyoD	complete	X16189	ENSGALG00000006216	MyoD
	*Xenopus tropicalis*	MyoD	complete	AJ579310	ENSXETG00000001320	MyoD
	*Xenopus laevis*	mf1	complete	M31116	n/a	MyoDa
		mf25	complete	M31118	n/a	MyoDb
**Ostariophysi**	*Danio rerio*	MyoD	complete	NM_131262	ENSDARG00000030110	MyoD1
	*Cyprinus carpio*	MyoD	complete	AB012882	n/a	MyoD1
	*Sternopygus macrurus*	MyoD	complete	AY396566	n/a	MyoD1
**Protacanthopterygii**	*Salmo salar*	MyoD1	complete	AJ557148	n/a	MyoD1a
		MyoD2	complete	AJ557149	n/a	MyoD1b
		MyoD1c	complete	DQ317527	n/a	MyoD1c
	*Oncorhynchus mykiss*	MyoD	complete	X75798	n/a	MyoD1a
		MyoD2	complete	Z46924	n/a	MyoD1b
		EST	partial	CX137438	n/a	MyoD1c
	*Salmo trutta*	MyoD1c	complete	DQ366710	n/a	MyoD1c
**Acanthopterygii**	*Takifugu rubripes*	MyoD1	complete	NM_001032769	SINFRUG00000154785	MyoD1
		MyoD2	complete	NM_001040062	SINFRUG00000163904	MyoD2
	*Tetraodon nigroviridis*	MyoD1	complete	AY616520	GSTENG00003954001	MyoD1
		MyoD2	fragmented	n/a	GSTENG00034775001	MyoD2
	*Oryzias latipes*	MyoD1	complete	n/a	ENSORLG00000000694	MyoD1
		MyoD2	fragmented	n/a	UTOLAPRE05100109983	MyoD2
	*Gasterosteus aculeatus*	MyoD1	complete	n/a	ENSGACG00000008444	MyoD1
		MyoD2	complete	n/a	ENSGACG00000017350	MyoD2
	*Sparus aurata*	MyoD1	complete	AY999688	n/a	MyoD1
		MyoD2	complete	AJ630127	n/a	MyoD2
	*Hippoglossus*	MyoD1	Partial	AF478568	n/a	MyoD1
	*hippoglossus*	MyoD2	complete	AF478569	n/a	MyoD2

## Results and Discussion

### Phylogenetic reconstruction of vertebrate MyoD sequences

**Figure 1:**
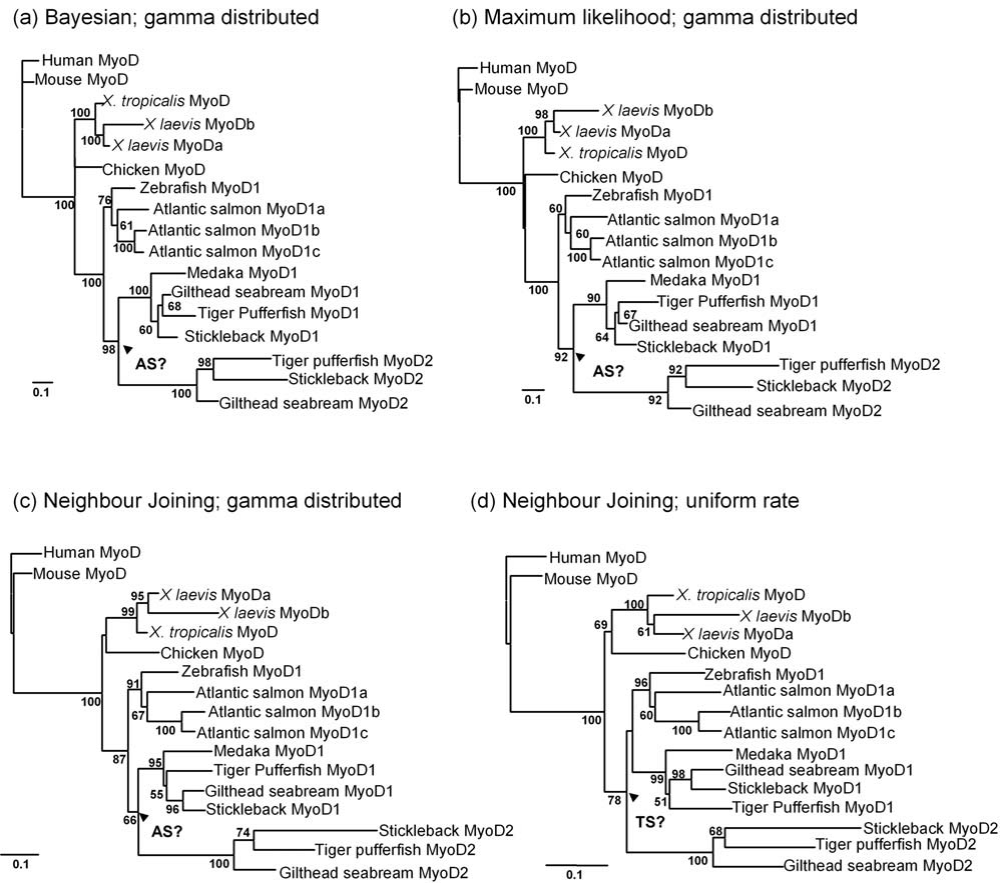
Unrooted phylograms of vertebrate MyoD amino acid sequences constructed using (a) Bayesian inference with a mixed model of amino acid substitutions and assuming a gamma distribution of among-site substitution rates (b) maximum likelihood with the WAG model of amino acid substitution and assuming a gamma distribution of among-site substitution rates (gamma distribution parameter estimated by PhyML to be 0.66) with 500 psuedobootstrap replicates for branch support (c) NJ with the Poisson correction model and assuming a gamma distribution of among-site rates (gamma distribution parameter = 0.66) and 1000 bootstrap replicates for branch support (d) NJ with the Poisson correction model assuming a uniform distribution of among-site substitutions rates with 1000 bootstrap replicates for branch support. Arrows marked AS refer to the Acanthopterygian specific (AS) positioning of the teleost MyoD1/2 duplication inferred in trees a–c. The arrow marked TS shows the teleost specific (TS) positioning of the teleost MyoD1/2 duplication event in tree d. Scale bars show the number of substitutions per site. Branch confidence values >50% from the different reconstruction methods are shown.

Our previous maximum likelihood (ML) and neighbour joining (NJ) tree based on amino acid translations of 62 MRFs, branched Acanthopterygian MyoD2 sequences externally to all vertebrate MyoD sequences [5]. This is an unexpected topology, and taken literally, suggests that MyoD2 arose in a common vertebrate ancestor prior to the separation of the Actinopterygii and Sarcopterygii and was then lost in all vertebrate lineages except the Acanthopterygian teleosts. A more expected position for this protein within a vertebrate MyoD tree topology would be to either branch from all teleost MyoD sequences, if it arose in a common teleost ancestor (e.g. during the teleost WGD), or from Acanthopterygian MyoD1 if a specific *myod* duplication occurred within this lineage as suggested previously [15]. We argue that this original tree topology is an artefact arising from the fact that Acanthopterygian MyoD2 has evolved asymmetrically relative to MyoD proteins in other vertebrate lineages (note the long branch lengths leading to Acanthopterygian MyoD2 sequences in Fig. 1a–d). Thus it is possible that the MyoD2 position was a consequence of long branch attraction (LBA) or mutational saturation, which are known problems in reconstructing phylogenetic relationships between orthologues and paralogues [16], [17].

Here we have produced a new MyoD alignment (Supplementary Information, Fig. S1), including paralogues found within different vertebrate lineages (salmonids, Acanthopterygians and frogs) but with reduced representation of potential long-branches, including sequences for Myf5, Mrf4 and Myog as well as basal-deuterostome MyoD orthologues (tunicate and amphioxus MyoD). By all methods of reconstruction, *X. laevis* MyoD paralogues, as expected, branched as a sister clade from *X. tropicalis* MyoD (Fig. 1a–d). As previously reported [5], all salmonid MyoD paralogues branched as co-orthologues of teleost MyoD1 (Fig. 1a–d). Bayesian, ML and NJ analyses placed the point of MyoD1/MyoD2 duplication as a specific event within the Acanthopterygii when a gamma distribution of among site rate variation was used which is known to be resistant to LBA [17] (Fig. 1a–c). Further, Bayesian and ML analyses also placed the duplication as a specific event to Acanthopterygians when among-site substitution rate variation was considered low or uniform (not shown). However, when a NJ tree analysis was performed assuming a uniform distribution of among site rate variation, the tree topology supported a common teleost origin of MyoD1/MyoD2 paralogues (Fig. 1d). Furthermore, by ‘pushing’ the gamma distribution parameter in PhyML to consider among-site rate variation as high-extremely high (performing the analysis with a gamma distribution parameter of 0.5, 0.4, 0.3, 0.2, 0.1, 0.01, 0.001) the resulting trees still supported an Acanthopterygian specific duplication event. Finally, by removing frequently mutating residues from the alignment before NJ tree reconstruction, a topology was retrieved supporting an Acanthopterygian-specific origin of paralogues (not shown). Thus, the majority of phylogenetic reconstructions performed with the new alignment (Fig. S1) clearly supported an Acanthopterygian specific event. Since the tree topology recorded in the previous phylogenetic analysis (which placed MyoD2 externally to all vertebrate MyoD sequences [5]) was not retrieved by any method employed here, it is possible that LBA may have affected the original tree reconstruction. Next, we used a comparative genomic approach to study the relationships of genes in neighbourhood to *myod* in several teleosts and two diploid tetrapods, expecting that some signal of *myod* duplication would be retrieved specifically in the Acanthopterygii.

### Synteny of vertebrate *myod* genes reveals the true extent of the teleost *myod1*/*myod2* duplication

**Figure 2:**
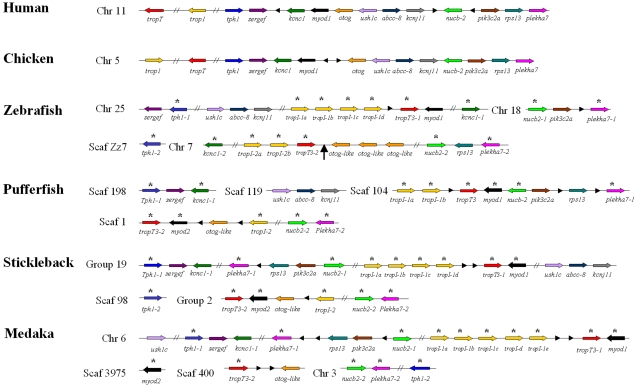
Diagram depicting the synteny conserved between the *myod*-containing chromosome of human, with that of chicken, zebrafish, pufferfish, stickleback and medaka. A striking pattern of interleaved double conserved synteny can be seen where teleost genes are distributed between two regions as either single copies or paralogues. This, in contrast to the direct depiction of MyoD phylogenetic relationships (Fig. 1), suggests that a *myod-*containing chromosome duplicated in a common teleost ancestor. Genes are not scaled by size and are represented by arrows (identifying the direction of transcription) coloured by their orthology to human genes. Black arrowheads represent genes not conserved between humans and other species on the chromosomal region investigated. Double diagonal lines represent a gap of more than three genes. Teleost genes found on the two paralogous chromosomal regions are marked with a black star. The black arrow on zebrafish chromosome 7 marks the putative position where *myod2* was non-functionalised. Teleost genes orthologous to those on zebrafish chromosomes 25 and 7 are respectively designated as Gene-1 and Gene-2, to identify their common paralogy. Multiple tandem *tropI* genes present on duplicated teleost chromosomes are labelled as *a, b, c* based on their left to right position and not by their inferred paralogy/orthology from phylogenetic reconstruction (Fig. 3d).

Our next aim involved establishing the chromosomal locations of genes in proximity to *myod* in human, relative to their positions in chicken, zebrafish and three Acanthopterygian species. This information was used construct a diagram of conserved synteny across the vertebrates (Fig. 2). Additionally, since *tropT* and *tropI* genes are in direct 3′ proximity to all teleost *myod* genes, we also assessed their location in human and chicken genomes. A very high degree of synteny is retained between the *myod* containing regions of human chromosome 11 and chicken chromosome 5 (Fig. 2). Comparing these regions with teleosts, while some inter and intra chromosomal rearrangements have occurred, a striking pattern of double conserved synteny (DCS) is observed where teleost genes are found as either single copies interspersed between two paralogous chromosomal tracts (*otog*, *abcc-8*, *kcnj11*, *pik3c2a*, *rps13*, *sergef*) or as at least two paralogues on both chromosomes (*tropT*, *tropI*, *tph1*, *kcnc1* [zebrafish specific], *nucb*2, *plekha7*) (Fig. 2). This pattern was maintained for genes found in both upstream and downstream proximity to *myod* in human/chicken and importantly, was observed in zebrafish (Ostariophysi) and the three Acanthopterygian species studied (Fig. 2). This common pattern of interleaved-DCS in teleosts is most consistent with the duplication of a *myod-*containing chromosome in a common ancestor to zebrafish (Ostariophysi) and the Acanthopterygii, but not tetrapods. We suggest that this occurred during the WGD of basal teleost evolution [9]. However, on zebrafish chromosome 5, the duplicated *myod2* gene is absent relative to its inferred position from Acanthopterygian genomes (Fig. 2, black arrow). The differential retention/loss of paralogues in different teleost lineages following the WGD is surprisingly common. For example, it was shown that ∼50% of zebrafish duplicates were retained as single copies in pufferfish genomes [18], [19]. Thus to summarise, the synteny conserved between *myod* neighbouring genes of tetrapods relative to teleosts strongly favours a teleost specific duplication of a *myod* containing chromosome in direct contradiction to the majority of topologies retrieved by direct phylogenetic reconstruction (e.g. Fig. 1).

### Phylogenetic reconstruction of *myod*-neighbouring genes supports synteny analysis

Next, we reconstructed the phylogenetic relationships of six genes found in proximity to *myod* in human/chicken genomes that were found as two copies on two paralogous chromosomes in teleosts. Amino acid sequences were aligned (alignments available on request to DJM) and analysed by Bayesian and ML methods. In 4/6 cases this approach immediately returned trees with topologies that were consistent with a common ancestry of teleost paralogues.

**Figure 3:**
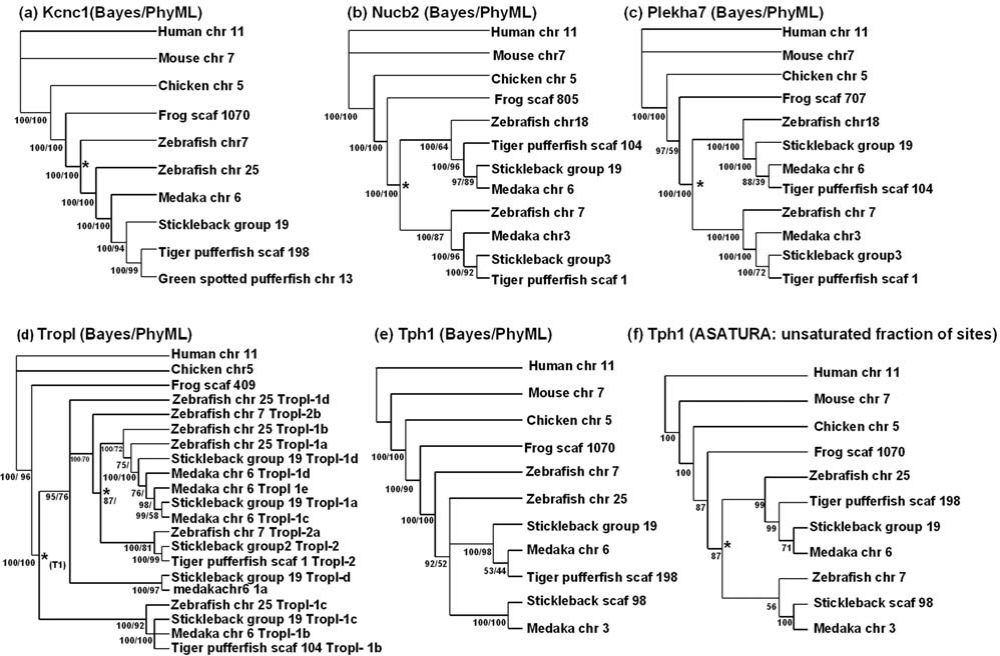
Unrooted phylogenetic cladograms for amino acid translations of genes in proximity to tetrapod *myod* that are conserved as two copies on two paralagous chromosomal regions in teleosts. Branch confidence values from different phylogenetic reconstruction methods are shown in the order they are bracketed. (a) Kcnc1 (Bayesian/ML topology). (b) Nucb2 (Bayesian/ML topology). (c) Plekha7 (Bayesian/ML topology). (d) TropI (Bayesian/ML topology). (e) Tph1 (Bayesian/ML topology). (f) Tph1 (topology corrected for mutational saturation). * represents a chromosomal duplication event arising in a common teleost ancestor. *_(T1) _represents the presumed first tandem duplication of *tropI*. Branch confidence values >50% from the different reconstruction methods are shown.

For Kcnc1, two copies were retained on the two paralagous chromosomes in zebrafish, but not Acanthopterygian species, which have retained this gene on a single chromosome orthologous to zebrafish chr 25 (Fig. 2). The Bayesian/ML analyses clustered one of the zebrafish paralogues (Kcnc1-1) with the Acanthopterygian sequences, and its paralogue, Kcnc1-2 (on chr 7) as an outgroup to these sequences, but internally to tetrapod orthologues (Fig. 3a). Nucb2 and Plekha7 paralogues, which are common to all teleosts examined (Fig. 2), formed two sister clades, each represented by sequences from all teleosts and branching from tetrapod orthologues (Fig. 3b–c). Thus, these three tree topologies supported a common teleost-specific origin for paralogues in each case with 100% Bayesian/ML branch confidence (black star on Fig. 3a–c)

Fast skeletal muscle specific *tropI* genes are closely associated with *myod* in all teleost genomes, and appear more distally downstream of *myod* in tetrapod genomes (Fig. 2). In teleosts, *trop1* can be found as distinct tandem paralogues (ranging from 2–5 in number) just downstream of *myod1*, but also in proximity to Acanthopterygian *myod2* genes and the position where the *myod2* gene of zebrafish was putatively lost (black arrow on chr 7, Fig. 2). Conversely, fast muscle specific *tropI* appears as a single gene on chromosomes 11 and 5 in human and chicken genomes. Thus, it seems that *tropI* has been though a series of in-chromosomal (tandem) duplications and a chromosomal duplication specifically during teleost evolution. For ease, we designated the tandem paralogues on each teleost chromosome as a, b, c etc, based solely on their left to right position on Fig. 2. To investigate their evolutionary relationships we reconstructed phylogenetic trees of all teleost TropI sequences within the scope of the synteny analysis using Bayesian and ML methods, which produced identical topologies (Fig. 3d). Interestingly, teleost sequences orthologous to zebrafish TropI-1c (stickleback-TropI-1c, medaka-TropI-1b, pufferfish-TropI-1b) clustered as an outgroup to all other teleost TropI sequences, with 100% branch support from both methods (Fig. 2d). This suggests that these TropI orthologues are the least derived relative to tetrapod TropI and are likely ancestral to all other teleost TropI paralogues, tandem or otherwise. The fact that the next teleost TropI sequences to branch internally to this clade (zebrafish TropI-1d, stickleback TropI-1d, medaka TropI-1a) are found on the same chromosome as the ‘ancestral’ TropI sequence, likely reflects an ancient tandem duplication event in a common teleost ancestor (Fig. 3,d). Internal to these braches, are TropI sequences from the paralogous chromosome (i.e. zebrafish chr 7, stickleback group 2 and tiger pufferfish scaf 1) (Fig. 3d). We argue that this branching reflects the chromosomal duplication event (black star on Fig. 3d) suggested by trees for other neighbouring genes (Fig. 3, a–c, f). Branches found internally to these sequences correspond to TropI sequences found in tandem with the ancestor TropI proteins (i.e. in zebrafish TropI-c and d). Thus to summarize, we suggest that *tropI* duplicated once in tandem prior to the teleost WGD event and other paralogues, either tandem or chromosomal are derived from these ancestral sequences. It is known that MyoD regulates the expression of fast muscle *tropI* genes through interactions with E-proteins [20]. The presence of multiple tandem fast-muscle *tropI* paralogues in close association with *myod* in teleosts but not tetrapods suggests that a selective advantage has arisen in teleost evolution for the tight regulation of multiple copies. Embryonic *in situ* expression data is available for one zebrafish fast skeletal muscle *tropI* gene. The zebrafish probe used by [21] (denoted *tnni2*) shares 100% identity to the putative Ensembl transcript of the *tropI-1d* gene (Fig. 2) and from mid-somitogenesis accumulated in muscles of the somite, fin buds and head [21] which overlaps spatially and temporally with *myod1* transcripts [22]. Additionally, in Atlantic cod (*Gadus morhua*) a cRNA probe orthologous to zebrafish *tropI-1d* was similarly expressed throughout the developing myotome during embryogenesis [23]. These findings suggest that this *tropI* gene is likely regulated by *myod1* during embryonic myogenesis. *In situ* expression data is not available for other fast-skeletal *tropI* genes. To gain insight into their regulation we performed tBLASTn searches of the EST database at GenBank using full amino acid translations of each zebrafish *tropI* gene within Fig. 2. A cut-off of 98–100% sequence identity was considered a positive hit from the returned sequences. Positive hits were returned for each *tropI* gene, confirming that each paralogue is transcribed into an mRNA product. Consistent with the *in situ* data, several hundred positive hits for zebrafish *tropI-1d* were retrieved solely from EST libraries representing embryonic zebrafish tissues. Interestingly, other *tropI* genes were not limited to embryonic tissues and were abundant in cDNA libraries obtained from adult zebrafish brain (*tropI-1c, 1a, 2a, 2b*), skin (*tropI-1c*), eye (*tropI-1b, 2a*), gill (*tropI-1c*), intestine (*tropI-1c*), gut (*tropI-1a*) and cultured myoblasts (*tropI-2b*). Similarly, BLAST searches of Atlantic salmon EST resources at the salmon genome project (http://www.salmongenome.no/) and the Gene Index Project (http://compbio.dfci.harvard.edu/tgi/) using the various zebrafish *tropI* amino acid sequences retrieved multiple salmon *trop-I* ESTs from tissue-specific cDNA libraries including fast muscle, slow muscle, gill, heart, skin, brain and eye. These findings suggest that the multiple ‘fast-muscle’ *tropI* paralogues found in teleosts are not solely involved in the assembly of fast skeletal muscle. Further their expression in multiple tissues is clearly not limited to regulation by muscle-specific transcription factors like *myod*. A more detailed examination of the expression patterns of teleost fast skeletal *tropI* duplicates would be a fruitful future experiment to gain insight into the evolution of *cis*-acting regulation of paralogues following gene duplication.

The Bayesian/ML trees retrieved for Tph1 and TropT paralogues, were not initially consistent with other trees and either branched one of the zebrafish genes as a sister group to its paralogue (TropT, not shown) or externally to all teleost genes (Tph1, Fig. 2e). These are possible artefacts arising from different rates of paralogue evolution between zebrafish and Acanthopterygian species. However, employing a gamma distribution of among-site rate variation in the Bayesian analysis did not change the topology of either tree, but did reduce posterior probability values at the suspected aberrant positions (not shown). To test for mutational saturation in these alignments we constructed NJ trees considering all substitution sites and then solely the unsaturated fraction of sites using ASATURA [16]. NJ trees considering all sites retrieved trees very similar to the Bayesian/ML analyses for both Tph1 and TropT (not shown). However, when the unsaturated alignments were analysed, both trees changed in topology, suggesting these alignments were affected by mutational saturation. The ‘unsaturated’ Tph1 NJ tree topology was changed in a manner consistent with other trees and supported a common origin of teleost paralogues (Fig. 2f). However, the expected topology was not retrieved for the TropT alignment by this approach and the two zebrafish sequences formed a sister clade with low branch confidence (not shown).

Thus to summarise, these supporting phylogenies are consistent with the synteny diagram (Fig. 2), and imply that a *myod* chromosome duplicated in a common teleost ancestor and again suggest that the position of the teleost MyoD1/MyoD2 duplication supported by direct phylogenetic analysis (Fig. 1) is almost certainly incorrect. These results highlight the importance of avoiding the use of single gene phylogenies when inferring the origin of gene paralogues and advocate the importance of studying the conserved synteny between, and phylogenetic relationships of, neighbouring genes in duplicated and non-duplicated lineages.

### Evolution of MyoD paralogues

**Figure 4:**
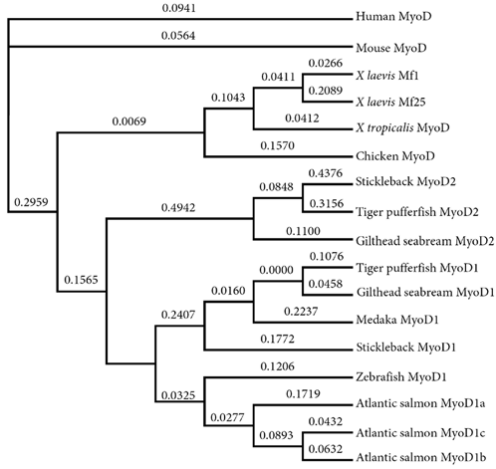
Unrooted ML cladogram of vertebrate MyoD amino acid sequences produced in PhyML [28] with an imposed ‘correct’ topology. The amino acid alignment was the same as used in Fig. 1. The imposed ‘correct’ starting tree topology supported the teleost WGD event (Acanthopterygii MyoD2 branching internally to tetrapod MyoD sequences, but externally to teleost MyoD1 sequences) and PhyML was used to refine branch lengths only. The ‘correct’ topology for other MyoD duplication events (in *X. Laevis* and Atlantic salmon) was as observed in trees in Fig. 1a–d. Branch lengths (substitutions per site) are shown above each branch.

To gain insight into the evolutionary rates of MyoD paralogues, a ML analysis was then performed, imposing the suggested correct topology of the teleost WGD (Acanthopterygian MyoD2 sequences branching internally to tetrapod MyoD orthologues but externally to teleost MyoD1 proteins: topology observed in Fig. 1d), but allowing the optimisation of branch lengths. The resulting cladogram and accompanying branch lengths can be seen in Fig. 4. Additionally, to examine differences in the evolutionary rates of MyoD paralogues and orthologues, we performed relative rate tests as described in the method section and shown in Table S1 (provided as supplementary information). *X. laevis* MyoD paralogues have clearly evolved asymmetrically and the branch length leading to Mf25 is around 8-fold greater than to Mf1 (Fig. 4). The relative rate test confirmed that Mf25 has evolved significantly faster than its paralogue (p = 0.002, not shown) with 24 unique substitutions relative to human MyoD compared to 7 for Mf1. Conversely, for Atlantic salmon MyoD1-co-orthologues, which are thought to have arisen from two salmonid-specific duplications of MyoD1 [5], differences in branch lengths are negligible (Fig. 4). Further, no significant differences in evolutionary rate were recorded between any two salmon MyoD1 co-orthologues in the relative rate test (Table S1). For Acanthopterygian MyoD paralogues, which almost certainly arose during the teleost WGD (see above, Fig. 2), asymmetric evolutionary rates were recorded as for *X. laevis*. The branch length in the Acanthopterygian MyoD2 lineage, prior to the separation of Gilthead seabream, pufferfish and sticklebacks is more than twice that of MyoD1 (Fig. 4). Additionally, evolutionary rates for individual stickleback and pufferfish MyoD2 sequences were strongly and significantly elevated compared to their MyoD1 paralogues (Fig. 4, Table S1). For example, the stickleback MyoD2 protein has 40 unique substitutions relative to human MyoD compared to 8 for MyoD1. Conversely, no significant difference in evolutionary rate was recorded between Gilthead seabream MyoD paralogues (Table S1). Interestingly, significant differences were also recorded in the evolutionary rate of MyoD2 orthologues when any two Acanthopterygian species were compared relative to human MyoD (Table S1). For example, the evolutionary rate of MyoD2 was respectively ∼4.5 and 2.5 times faster in stickleback than in Gilthead seabream and pufferfish (not shown). Conversely, differences in evolutionary rates between teleost MyoD1 orthologues were not significantly different except in one case when tiger pufferfish and zebrafish MyoD1 were compared (Table S1).

To summarise, there is strong evidence that differential evolutionary pressures exist on MyoD paralogues from different duplication events and whereas paralogues in *X. laevis* and Acanthopterygians have evolved asymmetrically, salmon duplicates have evolved at a comparable rate. Furthermore, species-specific rates of MyoD2 evolution were observed in the Acanthopterygii. This is consistent with a recent genome-wide study, where ∼600/2500 genes found to be present in the genomes of four different teleosts, showed significantly elevated or retarded rates of evolution in one of the teleost species compared to a human orthologue [24].

### A consensus nomenclature for vertebrate MyoD sequences

The genomic and phylogenetic results reported here provide strong evidence that a chromosomal region containing *myod* duplicated in a common teleost ancestor, but that *myod2* was lost in zebrafish (Fig. 2, Fig. 3). The absence of *myod2* genes in salmonid EST libraries [25] as well as minnow and catfish EST databases (DJM, unpublished result) suggests that this gene was also lost in the Ostariophysi and Protacanthopterygii lineages. The current vertebrate nomenclature is generally author-specific and based on the timing of MyoD discovery and does not account for evolutionary relationships of paralogues that have arisen in different vertebrate lineages. Our results allow us to advocate a consensus nomenclature relevant to all vertebrate *myod* genes. For teleost species that have arisen subsequent to the WGD, *myod* paralogues should be first identified by their orthology to either *myod1* or *myod2* and then more recently derived copies discovered within specific lineages should be named within this framework as *myod1(a/b/*etc*)* or *myod2*(*a/b/*etc). For other vertebrates that did not go through the teleost WGD, including tetrapods, and basal Actinopterygian groups such as the Acipenseridae and Lepisosteidae, *myod* orthologues retained as a single copy should be simply denoted *myod*, whereas lineage specific paralogues should be called *myoda*/*b*/etc (e.g. *X. laevis* MyoDa/b). This evolutionary relevant nomenclature, which is highlighted in Table 1, provides the simplest way of distinguishing between *myod* paralogues arising from the teleost WGD and those arising from lineage-specific duplication events. Furthermore, considering the frequency of polyploidy in fishes, amphibians and reptiles [10], [11] and the importance of the ongoing study of MyoD, it is likely that many more paralogues will be characterised in the future

## Materials and Methods

### Phylogenetic reconstruction of vertebrate MyoD sequences

Phylogenetic reconstruction of vertebrate MyoD was based on an alignment of amino acid translations of full-coding mRNA sequences from 17 vertebrate species (accession numbers/genbank IDs can be found in Table 1). These sequences were aligned with T-coffee [26] at (http://tcoffee.vital-it.ch/cgi-bin/Tcoffee/tcoffee_cgi/index.cgi) using a combination of Lalign and ClustalW alignments. Phylogenetic reconstruction was performed using Bayesian, maximum likelihood (ML) and neighbour joining (NJ) approaches. Bayesian analysis was performed in Mr Bayes 3.12 [27] with 2 parallel Metropolis-Coupled Markov Chain (MCMC) runs, four independent chains, a mixed amino acid model, sampling every 100 generations and assuming a gamma distribution of substitution rates. 500,000 generations were implemented with a burnin value corresponding to the first 150,000 generations. The runs were considered to have converged when the standard deviation of split frequencies was constantly less than 0.01 (this occurred after 150,000 generations) and trees from the burnin phase were discarded. A majority rule consensus tree was then built based on the final 3500 trees. A similar approach was also used without including a gamma distribution as a parameter. PhyML [28] was used to perform ML either with concurrent estimation of the gamma distribution parameter, or using a fixed value (see results and discussion text). The starting tree that was refined by ML was either the default BIONJ distance-tree or alternatively, a tree topology was uploaded (see results and discussion text), which was enforced while allowing optimisation of branch lengths. The WAG model (which gave the best posterior probability values in MrBayes) was employed with 500 pseudobootstrap replicates for branch confidence. NJ was performed in Mega 4 [29] using a gamma distribution of among site substitution rates (0.66, as estimated by PhyML), the Poisson correction model and 5000 bootstrap replicates. The same approach was also used to produce a NJ tree considering uniform among-site substitution rates. A NJ tree was also constructed considering solely the unsaturated fraction of substitution sites using ASATURA [16]. The WAG model was used and a cut off value of 2.584 was considered to remove saturated residues. Branch support was then obtained from 5000 bootstrap replicates.

### Synteny analysis of teleost *myod* genes

Genes in neighbourhood to human *myod* were manually obtained from the Ensembl database (www.ensembl.org) with the MultiContig View, Gene view and by using the orthologue/paralogue feature, while recording strand orientation and chromosomal position relative to *myod*. Orthologues of these genes were then obtained by the same approach for chicken (*Gallus gallus*), zebrafish (*Danio rerio*), pufferfish (*Takifugu rubripes*), Stickleback (*Gasterosteus aculeatus*) and medaka (*Oryzias latipes*) and a synteny diagram was constructed.

### Phylogenetic reconstruction of *myod*-neighbouring genes

Phylogenetic analysis was used to reconstruct the relationships of genes in upstream/downstream proximity to *myod* in human relative to other species used in the synteny analysis, and also using sequences obtained from Ensembl genomes databases of mouse (*Mus musculus*) and the diploid frog *X. tropicalis*. The criteria for gene selection was that two teleost copies were retained on two paralogous chromosomal regions, each retaining synteny to the single *myod*-containing chromosome of human/chicken genomes. Within our synteny analysis, this included genes coding for TropI, TropT, Kcnc1, Tph1, Nucb2 and Plekha7. High quality amino acid translations of these genes were manually obtained using the MultiContig/Geneview features at the Ensembl database. Sequences were aligned with T-coffee [26] at (http://tcoffee.vital-it.ch/cgi-bin/Tcoffee/tcoffee_cgi/index.cgi) using a combination of Lalign and ClustalW alignments. Phylogenetic reconstruction was performed using Bayesian and maximum likelihood (ML) approaches. Bayesian analysis was performed in Mr Bayes 3.12 [27] as described above. The number of generations and ‘burnin’ values for different sequences analysed were: TropI: 300,000 generations, burnin of 100,000 generations, TropT: 300,000 generations, burnin of 100,000 generations, Kcnc1: 100,000 generations, burnin of 25,000 generations, Tph1: 200,000 generations, burnin of 60,000 generations, Nucb2: 100,000 generations, burnin of 25,000 generations, Plekha7: 100,000 generations, burnin of 25,000 generations. Runs were considered to have converged when the average standard deviation of split frequencies between chains remained less than 0.01. Trees from the burnin phase were discarded and majority rule consensus trees and posterior probability values were calculated from trees obtained after runs had converged. ML was performed using PhyML [28], with the amino acid substitution model that gave the best posterior probability values in MrBayes (TropI: WAG, TropT: JTT, Kcnc1: JTT, Tph1: JTT, Nucb2: JTT, Plekha7: JTT), and assuming a gamma distribution of among-site substitution rates. 500 pseudobootstrap replicates were used to assess branch confidence. For TropT and Tph1, the tree topology returned by the Bayes/ML approach was inconsistent with the synteny/neighbouring genes analysis and trees retained for other *myod-*neighbouring genes. For these sequences we tested the hypothesis that mutational saturation may have affected the alignment. This was achieved in ASATURA [16], which was used to construct NJ trees with and without prior removal of frequently mutating residues from the alignment. The amino acid substitution with the highest MrBayes posterior probability values was used and branch confidence was assessed with 1000 bootstrap replicates. For the Tph1 alignment, the JTT matrix was employed and cut off values of 850 and 2348 were respectively used prior to tree reconstruction to consider all residues in the alignment and only the unsaturated fraction of sites. For the TropT alignment, the JTT matrix was employed and cut off values of 610 and 2258 were respectively used prior to tree reconstruction to consider all residues in the alignment and only the unsaturated fraction of sites.

### Relative rate test for MyoD sequences

To investigate whether MyoD paralogues and orthologues from different lineages evolved at different rates, Tajima's non-parametric relative rate test [30] was implemented in Mega 4.0 [29] based on amino acid sequences. For *X. laevis*, Mf1 and Mf25 paralogues were compared relative to the single MyoD orthologue of human. For each teleost species examined (zebrafish, Atlantic salmon, Tiger pufferfish, medaka, stickleback and Gilthead seabream) MyoD orthologues (or co-orthologues in the case of salmon) were compared relative to the human MyoD orthologue in all possible cross-species combinations, (e.g. zebrafish MyoD1 versus pufferfish MyoD1 compared to human MyoD). Similarly, all MyoD paralogues were compared within each teleost species relative to the human MyoD orthologue (e.g. MyoD1 versus MyoD2 in pufferfish compared to human MyoD).

## Supporting Information

Figure S1 Alignment of 17 vertebrate MyoD sequences at the amino acid level. The alignment was performed using T-coffee [26] with an initial input of Lalign and ClustalW alignments. Genbank accession numbers/Ensembl gene ID's can be found in Table 1 within the main text. The output is in the T-coffee format [26]. A colour scale can be found at the top of the alignment depicting sequence identities in a global context, as well as an overall ‘score’ for each sequence. Dashes indicate gaps in the alignment and stars highlight globally conserved residues, whereas colons and dots show conserved amino acid substitutions.
(0.06 MB DOC)

Table S1 Summary of non-parametric relative rate tests [30] performed at the amino acid level comparing various pairs of teleost MyoD paralogues and orthologues with the human orthologue of MyoD. The Chi-square (X) and p-values are shown. Abbreviations of teleost species are: Dr, Danio rerio, Ss, Salmo salar, Sa, Sparus aurata, Tr, Takifugu rubripes, Ga, Gasterosteus aculeatus and Ol, Oryzias latipes. Comparisons of MyoD orthologues are shown in black font. Comparisons of MyoD paralogues are shown in bold red font. A dash shows a comparison already recorded and N/A means not applicable.
(1.94 MB TIF)
