# Supplementary material for: An Update on MyoD Evolution in Teleosts and a Proposed Consensus Nomenclature to Accommodate the Tetraploidization of Different Vertebrate Genomes
Source: PLoS One. 2008 Feb 6;3(2):e1567. doi: 10.1371/journal.pone.0001567 (PMC2215776; doi:10.1371/journal.pone.0001567)
Supplement: Supplementary file 1 [file pone.0001567.s001.doc]

Supplementary Material Fig. S1

```
SCORE=71
*
 BAD AVG GOOD
*
Human MyoD                 :  67
Mouse MyoD                 :  69
X laevis MyoDb             :  68
X laevis MyoDa             :  73
Chicken MyoD               :  71
Zebrafish MyoD1            :  73
Atlantic salmon MyoD1a     :  72
Atlantic salmon MyoD1b     :  73
Atlantic salmon MyoD1c     :  73
Gilthead seabream MyoD1    :  73
Gilthead seabream MyoD2    :  68
Tiger pufferfish MyoD2     :  66
Tiger pufferfish MyoD1     :  72
Stickleback MyoD1          :  71
Stickleback MyoD2          :  64
Medaka MyoD1               :  71
X. tropicalis MyoD         :  72

Human MyoD                 MELLSPPLRD--VDLTAPDGSLCSFATTDDFYDDPCFDSPDLRFF
Mouse MyoD                 MELLSPPLRD--IDLTGPDGSLCSFETADDFYDDPCFDSPDLRFF
X laevis MyoDb             MELLPPALRD--MEET--EGSLCAFPTPDHFYDDPCFNSSDMSFF
X laevis MyoDa             MELLPPPLRD--MEVT--EGSLCAFPTPDDFYDDPCFNTSDMSFF
Chicken MyoD               MDLLGP------MEMT--EGSLCSFTAADDFYDDPCFNTSDMHFF
Zebrafish MyoD1            ------------MELS--DIPF-PIPSADDFYDDPCFNTNDMHFF
Atlantic salmon MyoD1a     ------------MELP--DIPF-PITSPDDFYDDPCFNTSDMHFF
Atlantic salmon MyoD1b     ------------MELS--DISF-PVTSADDFYDDPCFNTSDMHFF
Atlantic salmon MyoD1c     ------------MELS--DISF-PITSADDFYDDPCFNTSDMHFF
Gilthead seabream MyoD1    ------------MELS--DISF-PIPAADDFYDDPCFNTSDMHFF
Gilthead seabream MyoD2    ------------MDLS--DLPF-PLSSADDLYDDPCFSTSDMNFF
Tiger pufferfish MyoD2     ------------MDLS--ELVF-PL-SADDLYDDPCFSSGDLNFF
Tiger pufferfish MyoD1     ------------MELS--EISF-SIPAADDFYDDPCFSTSDMHFF
Stickleback MyoD1          ------------MELS--DISF-PIPAADDFYDDPCFDTSDMRFF
Stickleback MyoD2          ------------MDLP--DLSF-PLSSADDLYDDPCFSTGDMNFF
Medaka MyoD1               ------------MELS--DIPF-PIPAADDFYDDPCFNTSDMHFF
X. tropicalis MyoD         MELLPPPLRD--MEVT--EGSLCSFPTPDDFYDDPCFNTSDMSFF
cons                                   :: .  :  : .. :.*.:******.: *: **

Human MyoD                 EDLDPRLMHVGALLKPEEHSHFPA---------------AVHPAP
Mouse MyoD                 EDLDPRLVHMGALLKPEEHAHFPT---------------AVHPGP
X laevis MyoDb             EELEPRLVHVTLLKRGP--------------------------RH
X laevis MyoDa             EDLDPRLVHV-TLLKPEEPHH------------------------
Chicken MyoD               EDLDPRLVHVGGLLKPEEHPHTRA------------------PPR
Zebrafish MyoD1            EDLDPRLVHVS-LLKPDEHHH------------------------
Atlantic salmon MyoD1a     EDLDPRLVHVG-LLKPDDHHH------------------------
Atlantic salmon MyoD1b     EDLDPRLVHVG-LLKPDDHHY------------------------
Atlantic salmon MyoD1c     EDMDPRLVHVG-LLKPDDHHH------------------------
Gilthead seabream MyoD1    EDLDPWLVHVG-LLKPDDSSSSVSPSPSSSASSSPSSLLHLHHHA
Gilthead seabream MyoD2    DDLDARLMHAG-LLKPEDHLHHHH---------------HYHVPI
Tiger pufferfish MyoD2     DDLDSRL-----LLKPEGHQHLHHHVPSAEEE----------LEE
Tiger pufferfish MyoD1     EDMDPRLVHAG-LLKPDDCCSSSSLSPSSS-SASPSSLLHIHHHT
Stickleback MyoD1          EDLDPRLVHTG-LLKPDAP-----------------TLLHLHHRA
Stickleback MyoD2          DDLDSRLLQAG-LLKPDVP--------------------------
Medaka MyoD1               EDLDPRLVHGG-LLKP------------------------VHQRA
X. tropicalis MyoD         EDLDPRLVHV-ALLKPEDPHH------------------------
```

```
cons                        ::::. *     * :

Human MyoD                  GAREDEHVRAPSGHHQAGRCLLWACKACKRKTTNADRRKAATMRE
Mouse MyoD                  GAREDEHVRAPSGHHQAGRCLLWACKACKRKTTNADRRKAATMRE
X laevis MyoDb              EEEEEEHVRAPSGHHQAGRCLLWACKACKRKSSGADRRRAATMRE
X laevis MyoDa              --NEDEHVRAPSGHHQAGRCLLWACKACKRKTTNADRRKAATMRE
Chicken MyoD                EPTEEEHVRAPSGHHQAGRCLLWACKACKRKTTNADRRKAATMRE
Zebrafish MyoD1             --IEDEHVRAPSGHHQAGRCLLWACKACKRKTTNADRRKAATMRE
Atlantic salmon MyoD1a      --KGDEHIRAPSGHHQAGRCLLWACKACKRKTTNADRRKAATMRE
Atlantic salmon MyoD1b      --NEDEHIRAPSGHHQAGRCLLWACKACKRKTTNSDRRKAATMRE
Atlantic salmon MyoD1c      --NEDEHIRAPSGHHQAGRCLLWACKACKRKTTNTDRRKAATMRE
Gilthead seabream MyoD1     EGEDDEHVRAPSGHHQAGRCLLWACKACKRKTTNADRRKAATLRE
Gilthead seabream MyoD2     AEEEDEHVRAPGGLHQAGHCLLWACKACKRKTTHADRRKAATMRE
Tiger pufferfish MyoD2      ETVVEEHVRAPGGLHQAGRCLLWACKACKRKTTHADRRKAATMRE
Tiger pufferfish MyoD1      EAEDDEHIRAPSGHHHAGRCLLWACKACKRKTTNVDRRKAATLRE
Stickleback MyoD1           EAEDDEHVRAPSGHHQAGRCLLWACKACKRKTTNADRRKAATLRE
Stickleback MyoD2           --VLEGHVRAPGGLHQRGRCLLWACKACKRTTSLEDRRKAATMRE
Medaka MyoD1                EVEDDEHVRAPSGHHQAGRCLLWACKACKRKTTNADRRKAATLRE
X. tropicalis MyoD          --NEDEHVRAPSGHHQAGRCLLWACKACKRKTTNADRRKAATMRE
cons                            : *:***.* *: *:***********.::  ***:***:**

Human MyoD                  RRRLSKVNEAFETLKRCTSSNPNQRLPKVEILRNAIRYIEGLQAL
Mouse MyoD                  RRRLSKVNEAFETLKRCTSSNPNQRLPKVEILRNAIRYIEGLQAL
X laevis MyoDb              RRRLSKVNDAFETLKRCTSTNPNQRLPKVDILRNAISYIDSLQTL
X laevis MyoDa              RRRLSKVNEAFETLKRYTSTNPNQRLPKVEILRNAIRYIESLQAL
Chicken MyoD                RRRLSKVNEAFETLKRCTSTNPNQRLPKVEILRNAIRYIESLQAL
Zebrafish MyoD1             RRRLSKVNDAFETLKRCTSTNPNQRLPKVEILRNAISYIESLQAL
Atlantic salmon MyoD1a      RRRLSKVNDAFETLKRCTSTNPNQRLPKVDILRNAISYIESLQGL
Atlantic salmon MyoD1b      RRRLGKVNDAFENLKRCTSNNPNQRLPKVEILRNAISYIESLQSL
Atlantic salmon MyoD1c      RRRLGKVNDAFENLKRCTSNNPNQRLPKVEILRNAISYIESLQSL
Gilthead seabream MyoD1     RRRLSKVNDAFETLKRCTSANPNQRLPKVEILRNAISYIESPQAL
Gilthead seabream MyoD2     RRRLSRVNDAFETLKRCTASSPNQRLPKVDILRNAISYIESLQAL
Tiger pufferfish MyoD2      RRRLSKVNDAFETLKRCTASNPNQRLAKVEILRNAISYIESLQAL
Tiger pufferfish MyoD1      RRRLSKVNEAFETLKRCTNTNPNQRLPKVEILRNAISYIESLQAL
Stickleback MyoD1           RRRLSKVNDAFETLKRCTSANPNQRLPKVEILRNAISYIESLQAL
Stickleback MyoD2           RRRLGKVNDAFETLRRCTAAGPNRRLPKVEILRNAISYIESLQTL
Medaka MyoD1                RRRLSKVNDAFETLKRCTSANPNQRLPKVEILRNAISYIESLQAL
X. tropicalis MyoD          RRRLSKVNEAFETLKRCTSTNPNQRLPKVEILRNAIRYIESLQSL
cons                        ****.:**:***.*:* *  .**:**.**:****** **:. * *

Human MyoD                  LRDQ--DAAPPGAAAFYAPGPLPPGRGGEHYSGDSDASSPRSNCS
Mouse MyoD                  LRDQ--DAAPPGAAAFYAPGPLPPGRGSEHYSGDSDASSPRSNCS
X laevis MyoDb              LRDQ--EQS------------LYP--NMEHYSGDSDASSPSSNCS
X laevis MyoDa              LHDQ--DEA------FYP--------VLEHYSGDSDASSPRSNCS
Chicken MyoD                LREQ--EDA------YYP--------VLEHYSGESDASSPRSNCS
Zebrafish MyoD1             LRSQ--EDN------YYP--------VLEHYSGDSDASSPRSNCS
Atlantic salmon MyoD1a      LRGAGQEGN------YYP--------VMDHYSGDSDASSPRSNCS
Atlantic salmon MyoD1b      LRGQ-GGEN------YYP--------MLEHYSGDSDASSPQSNCS
Atlantic salmon MyoD1c      LRGQ-DGEN------YYP--------SLEHYNGDSDASSPRSNCS
Gilthead seabream MyoD1     LRG-GQDDG------YYP--------VLEHYSGDSDASSPRSNCS
Gilthead seabream MyoD2     LRT-GRDES------FYP--------PLEHYSGDSDASSPRSNCS
Tiger pufferfish MyoD2      LRTSGQDQS------FYP--------PLEPYGADSEASSPQSNCS
Tiger pufferfish MyoD1      LRG-GQDEA------FYT--------VLEHYSGDSDASSPRSNCS
Stickleback MyoD1           LRG-GQDDG------FYP-A------LLEHYSGDSDASSPRSNCS
Stickleback MyoD2           LGG----GG------FYP--------RRERHGEGSDACSPRSTCS
Medaka MyoD1                LRA-GQEDG------FYP--------VLEHYSGDSDASSPRSNCS
X. tropicalis MyoD          LRGQ--EES------FYP--------VLEHYSGDSDASSPRSNCS
cons                        *                           : :.  *:*.** *.**
```

```
Human MyoD                  DGMMDYSGPPSGARRRNCYEG-AYYNEAPSE-PRPGKS-AAVSSL
Mouse MyoD                  DGMMDYSGPPSGPRRQNGYDT-AYYSEAARE-SRPGKS-AAVSSL
X laevis MyoDb              DGM---NSPPCSSRRRNSYDS-NFYTDSPND-VRLGKS-SMISSL
X laevis MyoDa              DGMMDYNSPPCGSRRRNSYDS-SFYSDSPND-SRLGKS-SVISSL
Chicken MyoD                DGMMEYSGPPCSSRRRNSYDS-SYYTESPND-PKHGKS-SVVSSL
Zebrafish MyoD1             DGIMDFMGPTCQTRRRNSYDS-SYFNDTPNADARNNKN-SVVSSL
Atlantic salmon MyoD1a      DGMMDFNDPSCPPRRRNKYDS-IYFNETPND-SRRKKN-SVISSL
Atlantic salmon MyoD1b      DGMMDYNTPTCTSATRSNYYS-SYFAETPNAGARSNKNAAVISSL
Atlantic salmon MyoD1c      DGMMEYNAPTCTSARRSSYES-SYFAETPNADARSKKN-AVISSL
Gilthead seabream MyoD1     DGMTDFNGPSCQSNRRGSYDSSSYFSETPNGGLKSERS-SVVSSL
Gilthead seabream MyoD2     DGMMDFISP-CSSTSENS-DG-SFSNQTAYE-SRRSKR-SLVSSL
Tiger pufferfish MyoD2      DGAMDYVSPCVTSNAKNNRSR-RNIQTTGD---SSSKQ-CLVSSL
Tiger pufferfish MyoD1      DGMTDFNGPTCQSNRRGSYYS-SYFSQTPKGSLKAERN----SSL
Stickleback MyoD1           DGMTDFNGPTCQSNRRGSYDS-SYFSKTPNGGLKSHRS-SVVSSL
Stickleback MyoD2           EGPMDFISP-CSIRSENS-DGSYRIHTGPSG-TK---------PL
Medaka MyoD1                DGMTDFNGPRCQTTQKGSYDGGSYFSEALNGGHRNDRS-SVVSSL
X. tropicalis MyoD          DGMTDYS-PPCGSRRRNSYDS-SFYSDSPNG-LRLGKS-SVISSL
cons                        :*      *      ..                          .*

Human MyoD                  DYLSSIVERISTESPAAPALLLADV-PSE----SPPRRQEAAAP-
Mouse MyoD                  DCLSSIVERISTDSPAAPALLADA-PPE----SPPGPPEGASL-
X laevis MyoDb              DCLSSIVERISTQSPSCPAPISVDS-GSEG---SPCSPLQGETL-
X laevis MyoDa              DCLSSIVERISTQSPSCPVPTAVDS-GSEG---SPCSPLQGETL-
Chicken MyoD                DCLSSIVERISTDNSTCPILPPAEA-VAEG---SPCSPQEGGNL-
Zebrafish MyoD1             DCLSSIVERISTETPACPVLSVPE--GHEE---SPCSPHEGSVL-
Atlantic salmon MyoD1a      DCLSNIVERITTDTSACPAV--QD--GSEG--SSPCSPGDGSIA-
Atlantic salmon MyoD1b      DCLSNIVERIFTDTSASTVLSGQE--GSEG---SPCSPQEGSIL-
Atlantic salmon MyoD1c      DCLSSIVERISTDTSACTMLSV-----QEG---SPCSPQEGSIL-
Gilthead seabream MyoD1     DCLSSIVERISTDTSSLLPA--ADGP------ASPTTPPTGEAA-
Gilthead seabream MyoD2     DCLSSIVERISTDPAVAPPGDS----VVPQGPGSPQNSPTGSSP-
Tiger pufferfish MyoD2      ECLSSIVERISTDPTVVAPV--GDS-VVPR---GPGSPQ------
Tiger pufferfish MyoD1      DCLSSIVERISTATSSGPPP--VDGRGSPG---------------
Stickleback MyoD1           DCLSSIVERISTDSSSLQPA--SSASSTSGPGASPLDPPAAEAA-
Stickleback MyoD2           ACLSSIVERISTDPAVVPPGDS----VVPRGPGSPQSSPAGCG--
Medaka MyoD1                DCLSSIVERISTDSSCLPAT--ADAP------ASPAAAALSETER
X. tropicalis MyoD          DCLSSIVERISTESPVCPVIPAADS-GSEG---SPCSPLQGETL-
cons                        **.***** *  .

Human MyoD                  SEGESSGDPTQSPDAAPQCPAG-----ANPNPIYQVL
Mouse MyoD                  SDTE-QGTQTPSPDAAPQCPAG-----SNPNAIYQVL
X laevis MyoDb              SD---RGIPISSPGNSCTQLSH-----DPSSTIYQIL
X laevis MyoDa              SE---RVITIPSPSNTCTQLSQDPS-----STIYHVL
Chicken MyoD                SD---SGAQIPSPTN-CTPLPQESSSSSSNPIYQVL
Zebrafish MyoD1             SD---TGTTAPSPTS-CPQ-------QQAQETIYQVL
Atlantic salmon MyoD1a      SE---NGAPIPSPIN-CVPALHDP------NTIYQVL
Atlantic salmon MyoD1b      SE---TGAPVPSPTN-CPQPSHDP--------IYQVL
Atlantic salmon MyoD1c      SE---TGATVPSPTK-CPQPSHDP--------IYQVL
Gilthead seabream MyoD1     AP---GPVQIPSPT-----ASQDP------NLIYQVL
Gilthead seabream MyoD2     -----AGSS----------HPAE------PNSIYEPL
Tiger pufferfish MyoD2      ---------------------------SSPAGIYEPL
Tiger pufferfish MyoD1      -P-----LQASSPR-----SSREP------NLIYQVL
Stickleback MyoD1           AP-----VCVPSPT-----TSQDP------NLIYQVL
Stickleback MyoD2           ---------SSAGRN-RVEDSLSP------NKL----
Medaka MyoD1                GP---RAAPTSSPA-----SSQDP------NLIYQDL
X. tropicalis MyoD          SE---SGIIIPSPSNPCTHLSQDPS-----STIYQVL
cons                                                        :
```
